# Substitution of Fishmeal With Soy Protein Concentrate on Growth, Organic Damage, Antioxidant, Transcriptome, and Metabolomics in *Macrobrachium rosenbergii*

**DOI:** 10.1155/anu/6541143

**Published:** 2025-05-20

**Authors:** Li Wang, Qincheng Huang, Zhimin Gu, Cui Liu, Jia Xu, Yangxin Dai, Tiantian Ye, Junjun Yan, Jilun Meng, Yutong Zheng, Bo Liu

**Affiliations:** ^1^Biobreeding Institute, Xianghu Laboratory, Hangzhou 311231, Zhejiang, China; ^2^Wuxi Fisheries College, Nanjing Agricultural University, Wuxi 214081, Jiangsu, China; ^3^Guangxi Key Laboratory of Marine Environmental Science, Guangxi Academy of Marine Sciences, Guangxi Academy of Sciences, Nanning 530007, Guangxi, China; ^4^Institute of Fisheries Research, Hangzhou Academy of Agricultural Sciences, Hangzhou 310024, Zhejiang, China

**Keywords:** fishmeal, growth, *Macrobrachium rosenbergii*, soy protein concentrate

## Abstract

The present study investigated growth performance, body composition, hepatic and intestinal morphology, biochemical indices, transcriptomic responses, and metabolomic profiles in giant freshwater prawn (*Macrobrachium rosenbergii*) fed six kinds of soy protein concentrate (SPC) diets over an 8-week feeding trial. The six SPC diets were formulated by replacing varying proportions of fishmeal (FM) with SPC, with the final percentage of FM set at 350, 280, 210, 140, 70, and 0 g/kg, respectively, and designated as F35, F28, F21, F14, F7, and F0 diets. Results showed that the final body weight (FBW), weight gain, and specific growth rate (SGR) of prawn fed F35 were greater than those of prawn fed F7 and F0. The crude lipid content of prawn fed F0 was higher than that of prawn fed F28. Notably, increasing SPC substitution levels disrupted hepatopancreas morphology, with structural degradation becoming more pronounced beyond 40% replacement (F21). Compared to F35 group, the F0 significantly decreased the activity of glutathione peroxidase (GSH-PX), and increases the content of malondialdehyde (MDA) and nitric oxide (NO) in hemolymph. Based on the transcriptomics, two differentially expressed genes (DEGs) LOC136825138 and LOC136856310 were consistently observed across all groups. The metabolomics indicated that 77 differentially expressed metabolites (DEMs) across all treatments. A negative correlation was observed between LOC136856310 and eicosapentaenoic acid (EPA), arachidonoyl dopamine, 8Z,11Z-eicosadienoic acid, and vitamin E nicotinate. A comprehensive analysis of both metabolomic and transcriptomic data sets revealed substantial perturbations associated with “alpha-linolenic acid metabolism” and “glycerophospholipid metabolism”. In conclusion, elevated levels of dietary SPC had detrimental effects on the growth performance, hepatopancreas health, antioxidant capacity, and immune function of *M. rosenbergii*. Based on the growth performance, dietary FM level for *M. rosenbergii* could be reduced to 140 g/kg by using SPC as a sole substitute, with an inclusion of 211 g/kg of SPC in the diet.

## 1. Introduction

Fishmeal (FM) is regarded as one of the most important and extensively utilized protein sources in aquatic animal feed. This is due to its exceptional protein quality, well-balanced fatty acid and amino acid profiles, abundant vitamins and minerals, and excellent palatability [1] However, the declining availability of FM, caused by the overexploitation of pelagic fish stocks and the rapid expansion of the aquaculture industry, has made it essential to identify alternative protein sources [2]. Among the available alternatives, plant proteins have emerged as ideal substitutes for FM, not only because they lower feed costs but also because they help preserve wild fishery resources and mitigate environmental impacts [3]. Soybean is a prevalent plant protein source in aquafeed production, owing to its favorable amino acid profile, stable supply, and cost-effectiveness compared to FM [4, 5]. However, soybeans contain antinutritional factors (ANFs), which can hinder its utilization in meeting the nutritional demands of aquatic animals. These ANFs have been shown to impair a number of key processes, including feed intake, nutrient digestibility, and nutrient utilization. Furthermore, they have been demonstrated to exert a detrimental effect on disease resistance, which can consequently result in suboptimal growth and development in crustaceans [6].

In order to address these challenges, researchers have focused on bioprocessing soybeans into derivative products, such as soybean meal (SBM), soy protein isolate (SPI), fermented SBM (FSM), and soy protein concentrate (SPC) for use in aquatic animal diets [7, 8]. Among these, SPC, a product obtained through the leaching process of soybean flakes, is particularly notable for its reduced levels of ANFs and high protein content, approximately 65%, making it a promising alternative to FM [9]. Previous studies have demonstrated the potential of SPC as a partial substitute for FM in the diets of various aquatic species, including large yellow croaker (*Larimichthys crocea*) [2], olive flounder (*Paralichthys olivaceus*) [5], golden crucian carp (*Cyprinus carpio* × *Carassius auratus*) [10], pacific white shrimp (*Litopenaeus vannamei*) [4, 11], and tiger shrimp (*Penaeus monodon*) [12]. Nevertheless, the use of SPC is not without its limitations. For instance, residual ANFs, such as soybean antigen proteins, phytic acid, soybean agglutinin, and soya-saponins, have been identified as factors with the potential to exert a detrimental effect on animal health and performance [13–15]. Additionally, SPC contains comparatively lower levels of essential amino acids in comparison to FM, which poses another challenge for its use in aquafeeds [16]. Therefore, high levels of FM substitution with SPC have been shown to result in suboptimal growth performance and abnormal metabolic responses in aquatic species [10]. Consequently, it is imperative to ascertain the optimal level of SPC incorporation in aquafeeds, with a view to mitigating these deleterious effects while concomitantly maximizing its potential as FM substitute.

In recent years, transcriptomics and metabolomics have become important tools for studying nutrition and growth in aquatic animals. Transcriptomics offers a deeper understanding of how different nutrients, feed formulations, and environmental factors influence gene expression. Previous studies have used transcriptomic analysis revealed that SPC-induced enteritis in pearl gentian groupers (*Epinephelus fuscoguttatus* ♀ × *Epinephelus lanceolatus* ♂) may result from imbalances in intestinal nutrition and metabolism, which disrupt immune function, particularly affecting the IgA production pathway [17]. The comprehensive physiological and transcriptomic comparative analysis effectively evaluated the applicability of SPC as fish feed, offering valuable insights for designing an optimal diet to enhance growth performance and health in hybrid grouper [18]. On the other hand, metabolomics offers complementary information by profiling the metabolites involved in cellular processes, such as amino acids, lipids, and carbohydrates. It reveals how changes in the diet affect the metabolism of aquatic animals. Research has shown that different diets can significantly alter the metabolic profiles of fish and shrimp [19, 20]. These approaches provide a deeper understanding of how nutrition influences gene expression and metabolism in aquatic organisms, enhancing our knowledge of aquatic nutrition and helping to develop more sustainable and efficient feeding strategies in aquaculture.

The giant freshwater prawn (*Macrobrachium rosenbergii*), a globally cultivated species with significant economic value, is rapidly gaining prominence in aquaculture, particularly in China. In East China, a discernible shift has been observed from shrimp culture to *M. rosenbergii* farming, with the country's production reaching 196,400 tons in 2023, marking a 10.42% increase from the previous year [21]. The nutritional requirements of *M. rosenbergii* for optimal growth include a high-protein diet, typically containing approximately 40% protein, with FM serving as the primary ingredient [22]. This reliance on FM results in elevated feeding costs and limits the farming scale. Consequently, it is essential to identify sustainable alternatives to FM for *M. rosenbergii* feeds.

To the best of our knowledge, there is a paucity of studies evaluating the potential use of SPC in the diets of *M. rosenbergii* and its effects on prawn health and performance. Therefore, the present study aims to investigate the physiological, molecular, and metabolic responses of *M. rosenbergii* to varying levels of FM replacement with SPC. The assessment parameters encompass growth performance, proximate body composition, antioxidant capacity, immune responses, histopathological changes, transcriptomic analysis, and metabolomic profiling. The primary objective is to determine the optimal level of SPC substitution in *M. rosenbergii* diets, thereby reducing the dependance on FM while ensuring optimal growth and health outcomes. The findings are expected to contribute to the sustainable development of aquaculture by alleviating the pressure on global FM resources and ensuring the security of aquatic animal feeds.

## 2. Materials and Methods

### 2.1. Feed Ingredients and Formulation

The feed components utilized in this study comprised steam-dried FM, poultry by-product meal, SPC, dehulled SBM, wheat flour, soybean oil, and fish oil, all of which were sourced from Shanghai Fanyi Biotechnology Co., Ltd. (Shanghai, China). A comprehensive list of the primary feed ingredients can be found in Table 1.

In the reference diet (designated as F35), the FM content was set at 350 g/kg. Subsequently, diets F28, F21, F14, F7, and F0 featured 20%, 40%, 60%, 80%, and 100% replacement of FM with SPC, with the final percentage of FM set at 280, 210, 140, 70, and 0 g/kg, respectively. It is noteworthy that all six diets were formulated to maintain equal nitrogen content, with each containing approximately 390 g/kg of crude protein. Furthermore, these diets were isolipidic, with a lipid content of approximately 75 g/kg. The formulation and proximate composition of the experimental diets are detailed in Table 2, while the amino acid profiles are presented in Table 3.

In accordance with the methodology established by Sun et al. [22], all feed ingredients were subjected to a process of grinding through a 60 mm mesh, resulting in the creation of a fine powder. The powder was weighed with high precision prior to the incorporation of the lipid sources. The blend was then transferred to a 30 L kitchen mixer, where it was combined with a measured amount of tap water for a duration of 15 min. The pellets, approximately 1.5 mm in diameter and 3.0 mm in length, were then produced using a laboratory pelletizer from Shandong Saibainuo Machinery Co., Ltd. (Shandong, China). Thereafter, these diets were subjected to a drying process in a temperature-controlled environment at 25°C, after which they were stored in plastic bags in a refrigerator at −20°C until they were required for the experiments.

### 2.2. Prawns, Feeding, and Sampling

An experiment with a duration of 8 weeks was conducted at Zhejiang Lanke Seed Industry Technology Co., Ltd., situated in Huzhou, China. Prior to the initiation of the study, the *M. rosenbergii* underwent a 14-day acclimatization period where they were fed a commercial diet (feed no. 2, provided by Tongwei Feed Group Co., Ltd.) twice daily.

At the commencement of the experiment, the acclimated prawns were subjected to a 24-h fast. Twenty-four groups of 50 prawns each were collectively weighed and then divided into 24 tanks (measuring 97 × 57 × 61 cm). Each dietary treatment included four replicates. The initial mean body weight of the prawns was recorded as 0.22 ± 0.01 g (mean ± SD, *n* = 24). The feeding schedule involved the provision of sustenance to the prawns on two occasions per day, at 07:00 and 17:30, over a duration of 56 days (equivalent to 8 weeks). The quantity of feed provided ranged from 2% to 5% of the prawns' body weight. In order to ensure the water quality remained optimal, waste materials such as feces and shed exoskeletons were routinely siphoned from the tanks. Furthermore, 50% of the water in each tank was replaced on a daily basis with aerated pond water. Throughout the feeding trial, water temperatures varied between 25.1 and 30.9°C, and continuous aeration was implemented to keep the dissolved oxygen levels above 5 mg/L.

Subsequent to the termination of the experiment, the prawns were once more subjected to a 24-h period of fasting. Thereafter, individuals from each tank were meticulously gathered, weighed, and enumerated. From each tank, three prawns were randomly selected for assessment of body weight and hepatopancreas weight. These samples were then cryopreserved for subsequent proximate composition evaluations. Furthermore, hemolymph samples were pooled by combining the hemolymph from five prawns into a single sample, with three pooled samples collected per tank, totaling 15 prawns per tank. The hemolymph was collected using anticoagulant tubes that contained a sodium citrate solution and was subsequently subjected to centrifugation at 2000 × *g* for 10 min at a temperature of 4°C. The resulting serum was transferred into 1.5 mL cryogenic vials, which were then preserved in liquid nitrogen and subsequently stored at −80°C. Hepatopancreas and intestine samples were collected from six prawns per tank and stored at −80°C for subsequent analysis. For histopathological examination, four samples from both the hepatopancreas and intestine were collected per group and fixed in 4% paraformaldehyde, respectively. Additionally, four hepatopancreas samples per group were used for transcriptomic analysis. For metabolomic analysis, six hepatopancreas samples per group were collected from different prawns across the four replicate tanks to account for biological variability and enhance the reliability of untargeted metabolomics data.

### 2.3. Chemical Analysis

The proximate composition of both the experimental diets and body samples was analyzed in accordance with the methods specified by the Association of Official Analytical Chemists [23]. Specifically, the determination of crude protein was performed by calculating nitrogen content multiplied by a factor of 6.25, utilizing the Kjeldahl method after acid digestion. This process was facilitated by a Kjeltec 2300 Analyzer from Foss Tecator, a Swedish company. The assessment of crude lipid content was facilitated by the use of a SZF-06A fat extractor from Shanghai Xinjia Electronic Co., Ltd.. The moisture content was determined by subjecting the samples to oven-drying at 105°C until a constant weight was reached. Finally, the determination of crude ash content involved incinerating the samples in a muffle furnace at a temperature of 550°C until a stable weight was achieved.

### 2.4. Histological Observation

After fixation for more than 24 h, samples of hepatopancreas and intestines underwent dehydration using a series of increasing ethanol concentrations. Following this step, the samples were treated with xylene for equilibration and subsequently embedded in paraffin, in accordance with the methodology described by Ren et al. [24]. The paraffin-embedded tissues were carefully sliced into 4-µm-thick sections utilizing a rotary microtome (Leica Jung RM 2016, Germany). These sections were then stained with HE, with subsequent sealing and comprehensive drying. The prepared slides were observed under a microscope at a magnification of 200x using the ECLIPSE 90i model from Nikon, Japan, to facilitate thorough histological examination.

### 2.5. Biochemical Analysis

The present study quantitatively assessed the enzymatic activities of superoxide dismutase (SOD), glutathione peroxidase (GSH-PX), total antioxidant capacity (T-AOC), alkaline phosphatase (AKP), acid phosphatase (ACP) and the levels of malondialdehyde (MDA) and nitric oxide (NO) in hemolymph samples. These assessments were carried out with the help of assay kits provided by Nanjing Jiancheng Bioengineering Institute, China.

### 2.6. Transcriptome Analysis

Total RNA was extracted from the hepatopancreas samples with great care, and its quality and quantity were assessed in accordance with the methodology described by Xu et al. [25]. For the preparation of RNA samples, an initial quantity of 1 μg was utilized. The process of mRNA purification from the total RNA was achieved through the use of magnetic beads with poly-T oligonucleotides. The fragmentation of the mRNA was then performed with divalent cations in a specific fragmentation buffer supplied by Illumina. The synthesis of first-strand complementary DNA (cDNA) was then executed using random oligonucleotides in combination with Superscript II. The second-strand cDNA synthesis involved DNA polymerase I and RNase H, along with a treatment to convert any remaining overhangs into blunt ends through exonuclease/polymerase action. To enhance hybridization, an aptamer featuring a hairpin loop structure was subsequently ligated. Polymerase chain reaction (PCR) amplification was then conducted using Phusion High-Fidelity DNA polymerase, and the resultant products were purified by the AMPure XP system from Beckman Coulter, based in Beverly, USA. The integrity of the library was then evaluated on the Agilent Bioanalyzer 2100 system, developed by Agilent Technologies in Santa Clara, USA. Samples labeled with indexes were then clustered on the cBot Cluster Generation System using the TruSeq PE Cluster Kit v3-cBot-HS from Illumina, which is located in San Diego, USA. The finalized libraries were then subjected to sequencing on the NovaSeq 6000 platform, which was also produced by Illumina.

In order to ensure the highest possible quality of the clean reads, it was necessary to remove those sequences that contained adapters, poly-T elements, or were of low quality. The de novo assembly of the transcriptome was then carried out using Trinity software. The resulting unigenes were then aligned against the reference genome by HISAT2 version 2.0.5. For each gene, read counts were calculated to provide a measure of raw gene expression, followed by statistical analysis using HTSeq version 0.9.1. The differential expression analysis was executed with DESeq version 1.30.0, employing a statistical model based on a negative binomial distribution to pinpoint differentially expressed genes (DEGs). Genes that were identified as significantly differentially expressed had a false discovery rate (FDR) of less than 0.05 and a FC exceeding 2.00. Furthermore, enrichment analysis for Kyoto Encyclopedia of Genes and Genomes (KEGG) pathways related to these DEGs was performed using the ClusterProfiler R package, version 3.4.4, with pathways deemed significantly enriched when the *p*-value was less than 0.05.

### 2.7. Untargeted Metabolomics Analysis

For metabolomic profiling, the hepatopancreas was subjected to analysis. The analyses were conducted at Meiji Biomedical Technology Co. Ltd., a company based in Shanghai, China, which adhered to the LC–MS metabolomics methodology outlined by Cao et al. [26]. The extraction of metabolites was achieved by the addition of 400 μL of a solvent blend comprising methanol and water at a volume ratio of 4:1, which also included 0.02 mg/mL of the internal standard L-2-chlorophenylalanine. Mechanical disruption of the samples was carried out using a frozen tissue grinder, which was kept at −10°C and set to oscillate at a frequency of 50 Hz for a period of 6 min. Following this, cryosonic extraction was performed at 5°C for 30 min using an ultrasonic frequency of 40 kHz. Following this process, the samples were stored at −20°C for a further 30 min before undergoing centrifugation at 4°C for 15 min at 13,000 rpm. The resulting upper layer was then analyzed by the UPLC-Triple TOF system provided by SCIEX. Progenesis QI software from Waters Corporation in Milford, USA, was then utilized for the extraction of peaks, comparison, and retention time correction.

Mass spectrometry data and metabolic profiling information were obtained from several established public databases, such as the Human Metabolome Database (HMDB) and Metlin. These were then combined with a customized database matching strategy to enhance the accuracy of the analysis. In order to ensure the accuracy of the results, the peak areas were adjusted using the support vector regression (SVR) method, and log10 normalization was implemented on the generated data matrix. The subsequent analysis steps consisted of Principal Component Analysis (PCA) and Orthogonal Partial Least Squares Discriminant Analysis (OPLS-DA), both processed through version 1.6.2 of the R software package. The presence of differential metabolites was determined by calculating the significance scores of the projected variables (VIP) in the OPLS-DA model and combining them with the *p*-values obtained from the Student's *t*-test. Metabolites were identified as significantly different when their VIP values were greater than 1.00 and the *p*-value was less than 0.05. Finally, based on standard procedural protocols, these metabolites were mapped to metabolic pathways for association using the KEGG database.

### 2.8. Calculations and Statistics

The initial body weight (IBW), final body weight (FBW), weight gain, specific growth rate (SGR), survival rate, and hepatosomatic index (HSI) were calculated in accordance with the methodology proposed by Sun et al. [22]. The formulas are as follows:

  $$\begin{array}{c} \text{Weight Gain }\left(g\right)=\text{Final weight }\left(g\right)-\text{Initial weight }\left(g\right) \end{array}$$

  $$\begin{array}{c} \text{Specific Growth Rate }\left(\text{SGR},\%\;/\text{day}\right)=\left[\left(\text{Ln Final weight }-\text{Ln Initial weight}\right)|/|\text{ Time }|\left(\text{days}\right)\right]\times 100 \end{array}$$

  $$\begin{array}{c} \text{Survival Rate }\left(\%\;\right)=\left(\text{Number of surviving individuals }/\text{Total number of individuals at the start}\right)\times 100 \end{array}$$

  $$\begin{array}{c} \text{Hepatosomatic Index }\left(\text{HSI},\%\right)=\left(\text{Hepatopancreas weight }\left(g\right)/\text{Body weight }\left(g\right)\right)\times 100 \end{array}$$

To assess the effect of FM replacement level on the aforementioned indices, a one-way ANOVA was performed focusing on survival rate, FBW, weight gain, SGR, HSI, whole-body composition, SOD, GSH-PX, T-AOC, AKP, and ACP activities, as well as MDA and NO concentrations in hemolymph. To analyze the differences between F35, F28, F21, F14, F7, and F0, the Duncan test was employed. All statistical analyses were performed using SPSS software (version 24.0, SPSS Inc., USA), and the significance level was set at *p* < 0.05.

## 3. Results

### 3.1. The Effects of SPC Substitution on Growth Performance and Proximate Composition

During the harvest phase, survival rates for the F35, F28, F21, F14, F7, and F0 groups were documented as 70%, 78%, 75%, 73%, 80% and 72%, respectively (Table 4). FBW, weight gain, and SGR were dependent (*p* < 0.05), while IBW and survival were independent (*p* > 0.05), on FM replacement level. No significant differences between all groups in terms of IBW and survival (*p* > 0.05). The FBW and weight gain of prawn fed F35 were greater than those of prawn fed F7 and F0 (*p* < 0.05), but were not significantly different from those of prawn fed F28, F21, and F14 (*p* > 0.05).

Crude lipid and ash content were dependent (*p* < 0.05), while HSI, moisture content, and crude protein were independent (*p* > 0.05), on FM replacement level (Table 5). No significant differences were found in HSI, moisture content and crude protein among all groups (*p* > 0.05). The crude lipid content of prawn fed F0 was greater than that of prawn fed F28 (*p* < 0.05), but was not significantly different from those fed F35, F21, F14, and F7 (*p* > 0.05). The ash content of prawns fed F14 was lower than that of prawns fed F7 (*p* < 0.05), but did not significantly differ from those fed F35, F28, F21, and F0 (*p* > 0.05).

### 3.2. The Effects of SPC Substitution on Hepatopancreas and Intestinal Morphology

**Figure 1:**
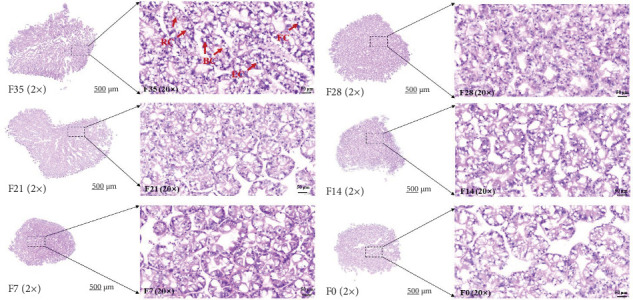
The morphology of hepatopancreas of *M. rosenbergii* fed the test diets. ECs: embryonic cells; BCs: secretory cells; FCs: absorptive cells; RCs: storage cells. F35: reference diet; F28, F21, F14, F7, and F0: soy protein concentrate replaced 20%, 40%, 60%, 80%, and 100% of the fish meal in diet F35, respectively.

In the F35 and F28 groups, hepatopancreatic cells exhibited intact basement membranes with well-defined margins, and a substantial number of embryonic cells (ECs) were observed. Some ECs were dispersed within the lumen (Figure 1). Secretory cells (BCs) and storage cells (RCs) were present in high abundance, occasionally forming bilayered structures. The F21 group maintained an intact stromal membrane; however, cell margins appeared less distinct, resulting in poorly defined boundaries. RCs counts were low, accompanied by a slight decrease in the number of swollen BCs. In contrast, hepatopancreatic cells in the F14, F7, and F0 groups displayed disrupted basement membranes with a pasty appearance. A notable reduction in the number of both BCs and RCs was observed, with BCs exhibiting significant swelling.

**Figure 2:**
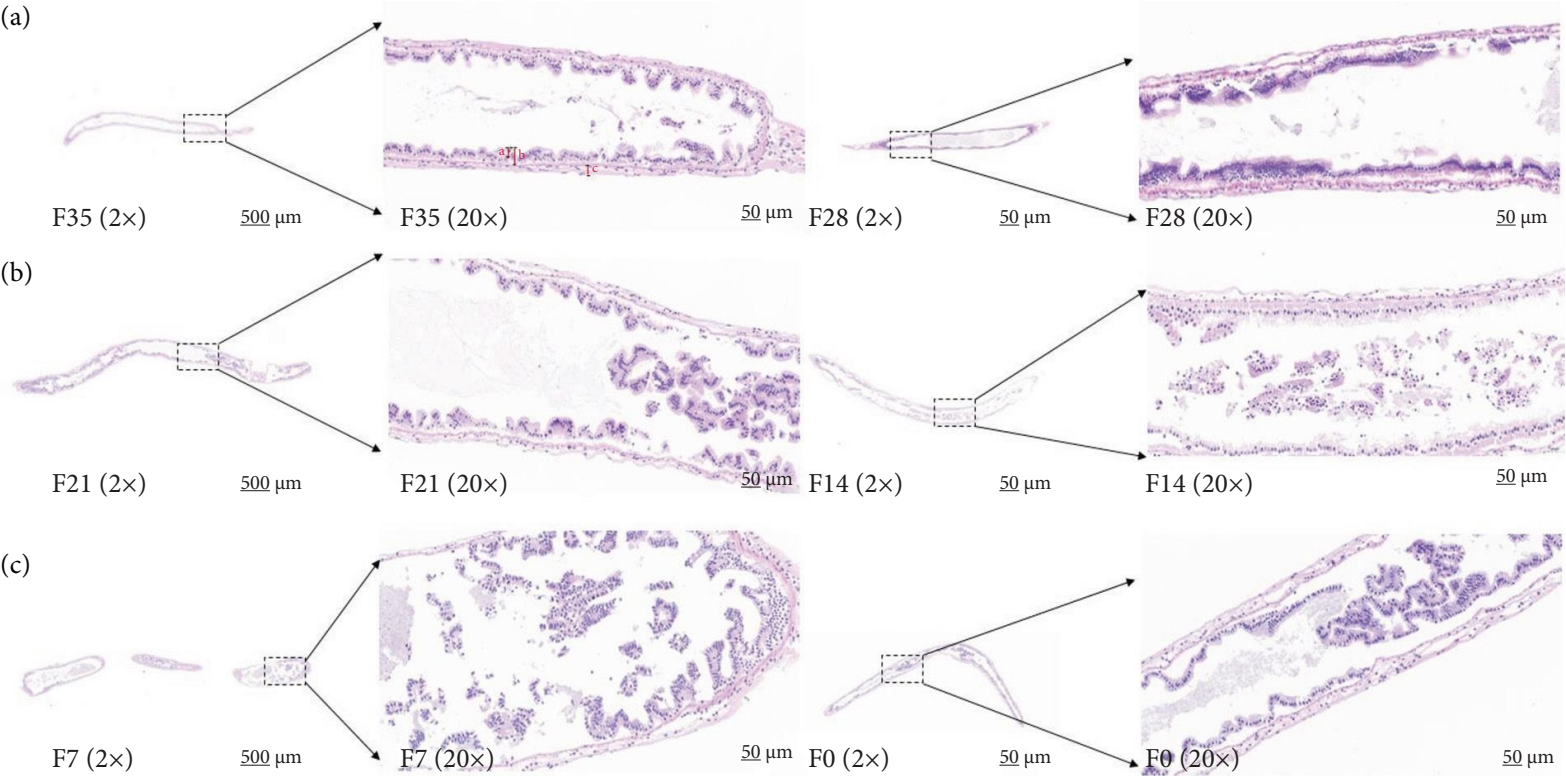
The morphology of intestine of *M. rosenbergii* fed the test diets. (a) Fold height, (b) mucosal thickness, and (c) muscular thickness. F35: reference diet; F28, F21, F14, F7, and F0: soy protein concentrate replaced 20%, 40%, 60%, 80%, and 100% of the fish meal in diet F35, respectively.

No significant differences were detected between all groups regarding the activities of fold height and muscular thickness in intestinal sections (*p* > 0.05) (Figure 2 and Table 6). Notably, the F21 treatment exhibited the highest mucosal thickness, followed by the F28, F7, F14 and F35 treatments, with the F0 treatment showing the lowest values (*p* < 0.05).

### 3.3. The Effects of SPC Substitution on Biochemical Indices

The activities of T-AOC, SOD and ACP showed no significant differences between the F35, F28, F21, F14, F7 and F0 groups (*p* > 0.05) (Table 7). The GSH-PX activity of prawns fed F35 and F28 was higher than that of prawns fed F7 and F0 (*p* < 0.05), but did not significantly differ from those fed F21 and F14 (*p* > 0.05). The MDA content of prawns fed F0 was higher than that of prawns fed F14 (*p* < 0.05), but did not significantly differ from those fed F35, F28, F21, and F7 (*p* > 0.05). The AKP activity of prawns fed F0 was higher than that of prawns fed F35, F28, F21, F14, and F7 (*p* < 0.05). The NO content of prawns fed F0 was higher than that of prawns fed F35, F28, F21, F14, and F7 (*p* < 0.05).

### 3.4. The Effects of SPC Substitution on Hepatopancreas Transcriptome

**Figure 3:**
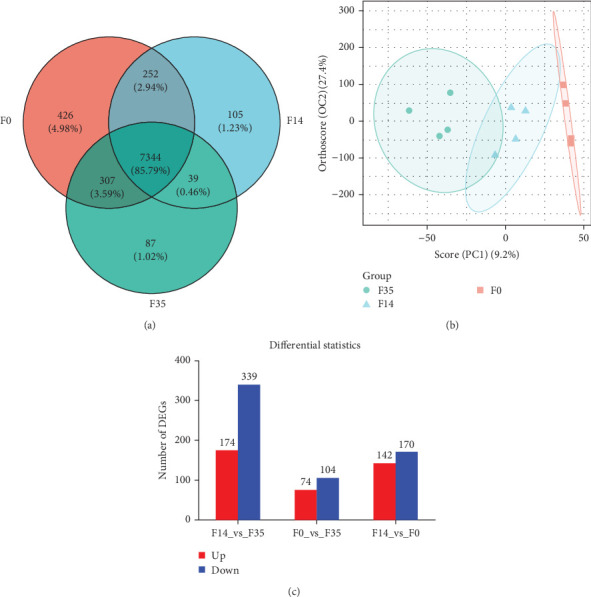
The VN plot analysis (A), OPLS-DA analysis (B), and numbers of differentially expressed genes (C) of *M. rosenbergii* fed the test diets. The VN plots and OPLS-DA analyses were based on data pertaining to the annotated gene set. Genes with significant differential expression were those having a false discovery rate (FDR) less than 0.05 and a fold change greater than 2.00. F35: reference diet; F14 and F0: soy protein concentrate replaced 60% and 100% of the fish meal in diet F35, respectively.

The RNA-seq analysis of the hepatopancreas produced an average of 4.67◊10^7^ raw reads. Following optimization and quality control procedures, there were approximately 4.63◊10^7^ clean reads and 6.91◊10^9^ clean bases, yielding an average clean rate of 98.77%. Upon aligning with the *M. rosenbergii* genome (GCF_040412425.1), an average of 4.63◊10^7^ reads were mapped, resulting in a mapping rate of 94.54%. The VN plot analysis indicated that a significant majority of sequences obtained through high-throughput screening were shared, approximately 7344 sequences, which accounted for about 85.79% of the total (Figure 3A). The OPLS-DA analyses showed that the samples from F35 and F14 groups were generally distinguishable and distributed in different directions, but there was a small overlap, and there was also a partial overlap between the distributions of samples from F14 and F0 groups (Figure 3B). In contrast, the samples of F35 and F0 groups were clustered separately. This suggests that the structural composition of expressed genes in prawn gradually changed with increasing levels of substituted FM.

In the F14 group, compared to the F35 group, 513 DEGs were identified, comprising 174 upregulated and 339 downregulated genes (Figure 3C). In the F0 group, relative to the F35 group, 178 DEGs were identified, including 74 upregulated and 104 downregulated genes. In the F0 group, 312 DEGs were observed compared to the F14 group, consisting of 142 upregulated and 170 downregulated genes. Across all experimental groups, two DEGs were consistently observed: LOC136825138 (uncharacterized lncRNA) and LOC136856310 (sulfinoalanine decarboxylase).

**Figure 4:**
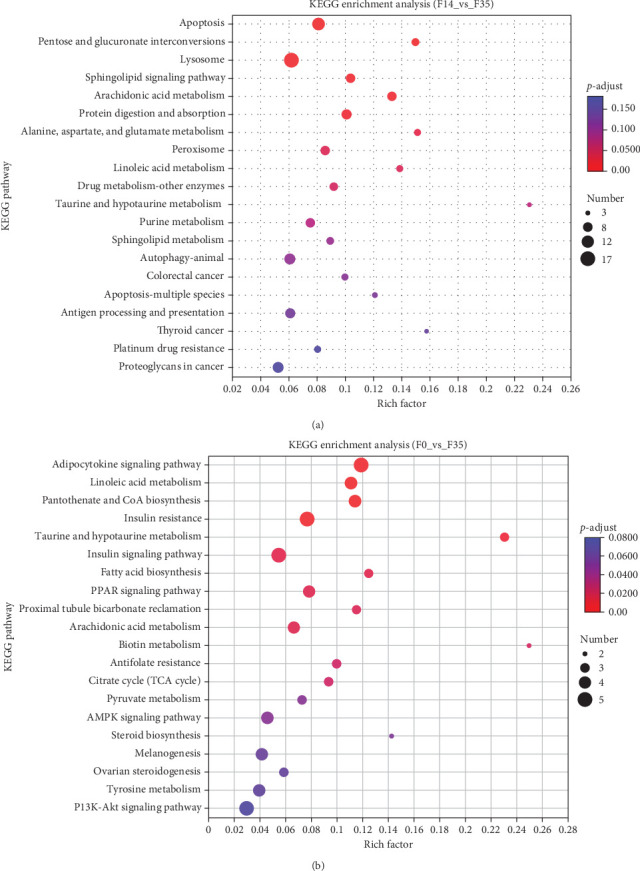
Enrichment analysis of KEGG pathways basing on transcriptome between *M. rosenbergii* fed F14 vs F35 groups (A) and F0 vs F35 groups (B). Pathways were considered significantly enriched when the *p*-value was below 0.05. F35: reference diet; F14 and F0: soy protein concentrate replaced 60% and 100% of the fish meal in diet F35, respectively.

Database comparison revealed that the significantly enriched KEGG pathways in the F14 group, relative to the F35 group, were “apoptosis”, “pentose and glucuronate interconversions”, “lysosome”, “sphingolipid signaling pathway”, “arachidonic acid metabolism”, “protein digestion and absorption”, “alanine, aspartate, and glutamate metabolism”, “peroxisome” and “linoleic acid metabolism” (Figure 4A). For the F0 group, compared to F35 group, the significantly enriched pathways were “adipocytokine signaling pathway”, ”linoleic acid metabolism”, “pantothenate and CoA biosynthesis”, “insulin resistance”, “taurine and hypotaurine metabolism”, “insulin signaling pathway”, “fatty acid biosynthesis”, “PPAR signaling pathway”, “proximal tubule bicarbonate reclamation”, “arachidonic acid metabolism”, “biotin metabolism”, “antifolate resistance”, “citrate cycle (TCA cycle)”, “pyruvate metabolism” and “AMPK signaling pathway” (Figure 4B). Furthermore, two KEGG pathways were shared, involving “linoleic acid metabolism” and “arachidonic acid metabolism”.

### 3.5. The Effects of SPC Substitution on Hepatopancreas Metabolomics

**Figure 5:**
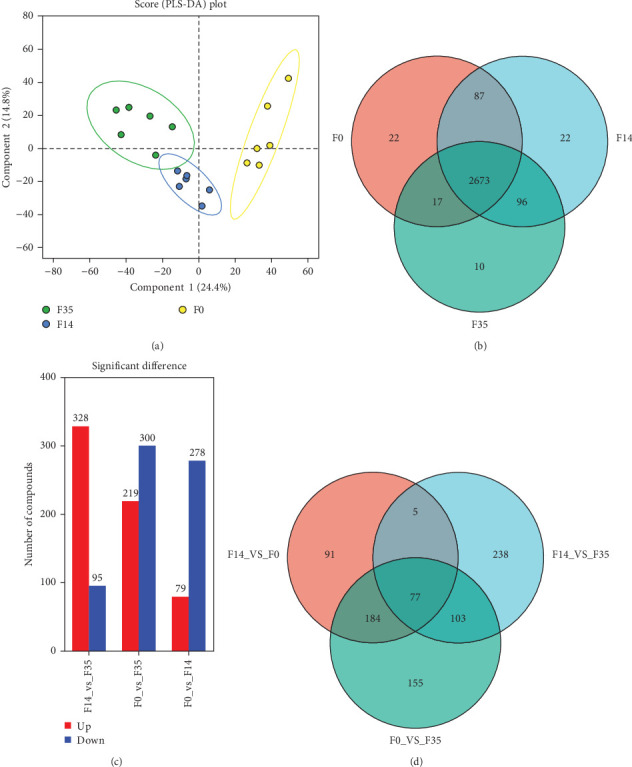
The PLS-DA (A) and VN plot (B) analysis of metabolites, and the numbers (C) and VN plot (D) of differentially expressed metabolites of *M. rosenbergii* fed the test diets. Metabolites were considered significantly differential if they exhibited variable importance in projection (VIP) values exceeding 1.00 and *p*-values below 0.05. F35: reference diet; F14 and F0: soy protein concentrate replaced 60% and 100% of the fish meal in diet F35, respectively.

The impact of SPC substitution on hepatopancreas metabolomics was investigated using LC-MS analysis. In the PLS-DA analysis of metabolites (Figure 5A), Component 1 (C1) accounted for 24.4% of the total variance, whereas Component 2 (C2) explained 14.8%. The analysis effectively distinguished the samples from the F35 and F14 groups, despite a minor overlap. Notably, significant clustering of F35 and F14 group samples occurred along the C1 axis, with no substantial clustering along the C2 axis, indicating that C1 was primarily responsible for differentiating these two groups. With increasing SPC substitution levels, the distribution of F0 group samples diverged from the F35 group, F0 and F35 groups were distinctly separated. The VN plot of metabolites revealed that 2,673 metabolites were shared across all experimental groups, constituting approximately 91.32% of the total (Figure 5B).

**Figure 6:**
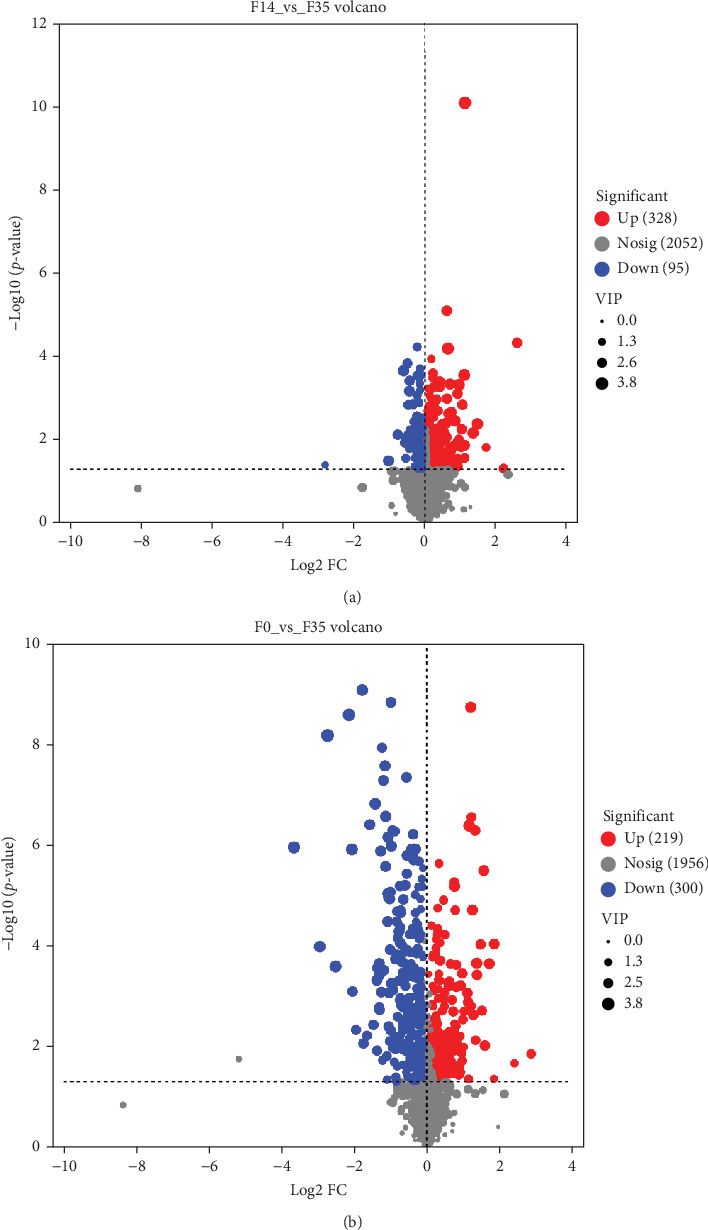
Volcano map of differentially expressed metabolites between *M. rosenbergii* fed F14 vs F35 groups (A) and F0 vs F35 groups (B). Metabolites were considered significantly differential if they exhibited variable importance in projection (VIP) values exceeding 1.00 and *p*-values below 0.05. F35: reference diet; F14 and F0: soy protein concentrate replaced 60% and 100% of the fish meal in diet F35, respectively.

**Figure 7:**
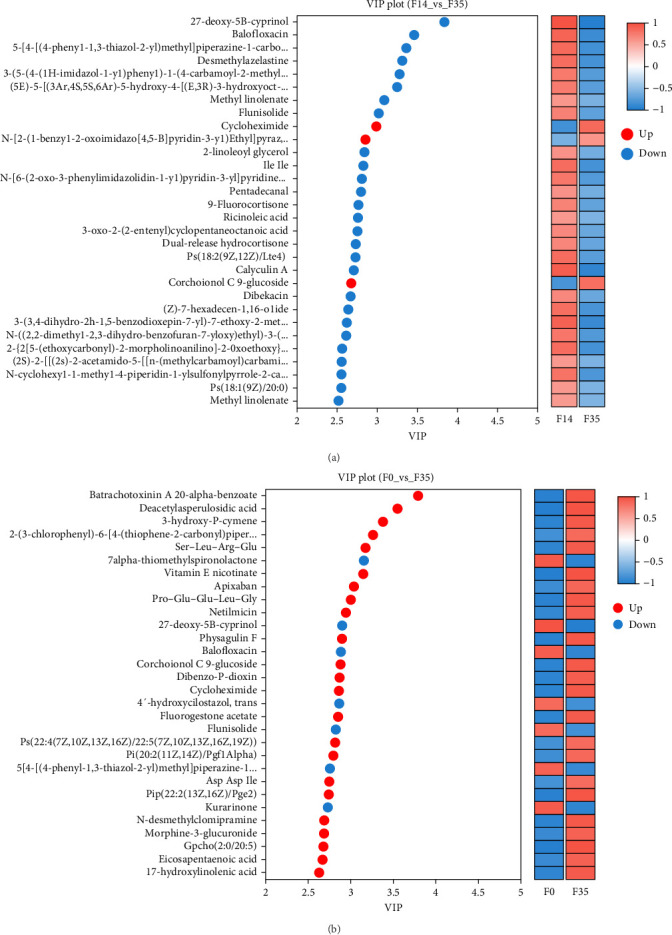
Variable importance in projection (VIP) of differentially expressed metabolites between *M. rosenbergii* fed F14 vs F35 groups (A) and F0 vs F35 groups (B). Metabolites were considered significantly differential if they exhibited variable importance in projection (VIP) values exceeding 1.00 and *p*-values below 0.05. F35: reference diet; F14 and F0: soy protein concentrate replaced 60% and 100% of the fish meal in diet F35, respectively.

In comparison to the F35 group, the F14 treatment exhibited 423 differentially expressed metabolites (DEMs), comprising 95 downregulated and 328 upregulated metabolites (Figures 5C and 6). The F0 treatment, relative to the F35 group, revealed 519 DEMs, including 300 downregulated and 219 upregulated metabolites. In the F0 group, 357 DEMs were observed compared to the F14 group, consisting of 79 upregulated and 278 downregulated metabolites. The VN plot of DEMs identified 77 common DEMs across all groups (Figure 5D). Variable projected importance (VIP) analysis, utilizing C1 in PLS-DA, pinpointed significant metabolites influencing classification (Figure 7). Between the F14 and F35 groups, the top three VIP values corresponded to 27-deoxy-5B-cyprinol, balofloxacin, and 5-[4-[(4-phenyl-1,3-thiazol-2-yl) Methyl] piperazine-1-carbonyl] pyran-2-one. For the F0 group, compared to F35 group, the foremost VIP metabolites were batrachotoxinin A 20-alpha-benzoate, deacetylasperulosidic acid, and 3-hydroxy-P-cymene. Furthermore, the six VIP metabolites were consistently observed across all groups: 27-deoxy-5B-cyprinol, balofloxacin, 5-[4-[ (4-phenyl-1,3-thiazol-2-yl) methyl] piperazine-1-carbonyl] pyran-2-one, flunisolide, cycloheximide, and corchoionol C 9-glucoside.

**Figure 8:**
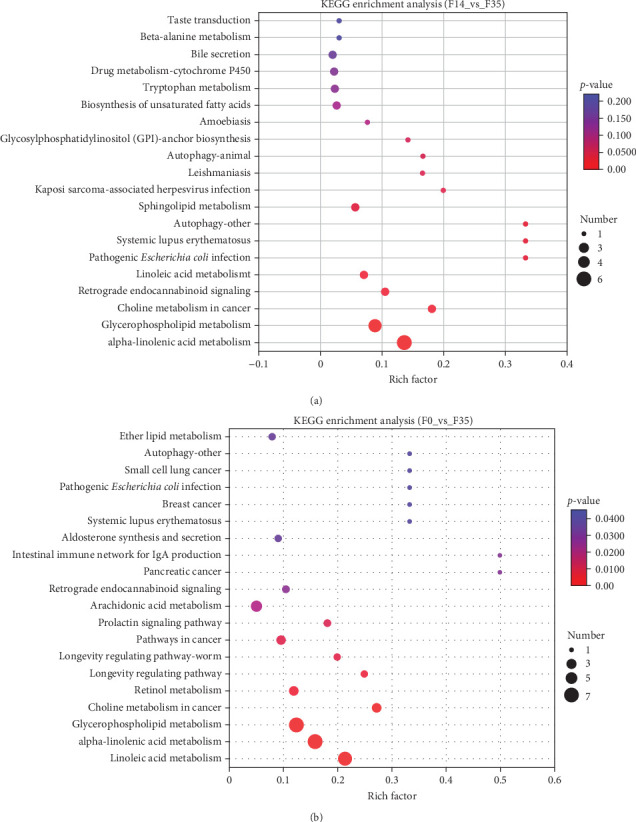
Enrichment analysis of KEGG pathways basing on metabolomics between *M. rosenbergii* fed F14 vs F35 groups (A) and F0 vs F35 groups (B). Pathways were considered significantly enriched when the *p*-value was below 0.05. F35: reference diet; F14 and F0: soy protein concentrate replaced 60% and 100% of the fish meal in diet F35, respectively.

Moreover, predicting the prevalence of KEGG functional pathways based on DEMs was accomplished using PICRUSt2 across each cohort. The most significantly enriched pathways between the two groups (F14 vs F35 and F0 vs F35) were demonstrated in Figure 8. Across all treatment groups, the six KEGG pathways “glutathione metabolism”, “kaposi sarcoma-associated herpesvirus infection”, “autophagy-other”, “autophagy-animal”, “primary bile acid biosynthesis”, and “purine metabolism” were consistently enriched at the third category level.

### 3.6. The Integration of Transcriptome and Metabolome Analyses in Hepatopancreas

**Figure 9:**
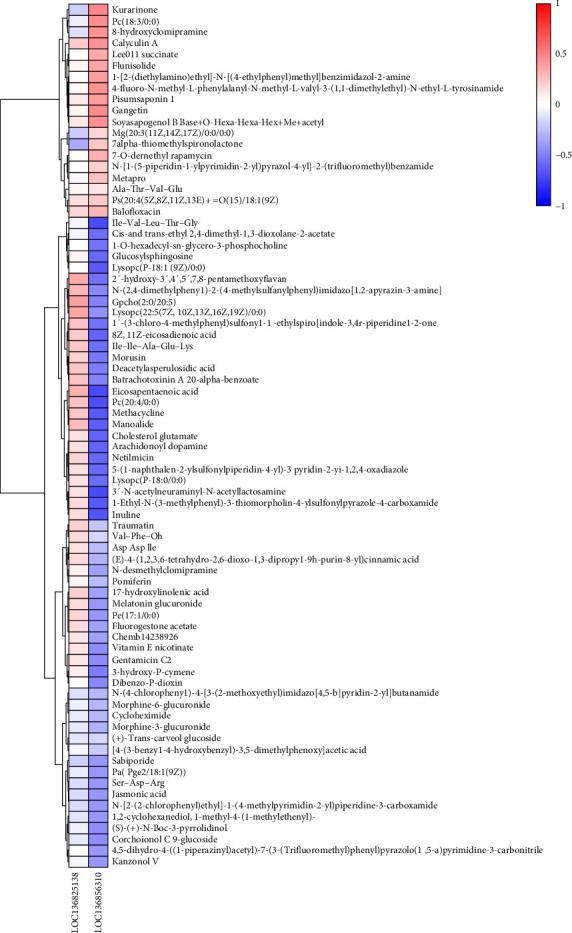
The spearman correlation analysis of differentially expressed genes and differentially expressed metabolites across F35, F14, and F0 groups. Red indicates a positive correlation and blue indicates a negative correlation. F35: reference diet; F14 and F0: soy protein concentrate replaced 60% and 100% of the fish meal in diet F35, respectively.

In order to ascertain the correlation between fluctuations in the DEGs and the profiles of DEMs susceptible to SPC substitution, Spearman correlation analysis was conducted. Consequently, a correlation analysis was performed on 2 DEGs and 77 DEMs that were present in all treatment groups, and the results are presented in a heatmap (Figure 9). Furthermore, we find that the LOC136856310 (sulfinoalanine decarboxylase) was significantly negatively corrected with the eicosapentaenoic acid (EPA), inuline, arachidonoyl dopamine, 8Z,11Z-eicosadienoic acid, and vitamin E nicotinate, whereas significantly positively corrected with soyasapogenol B Base+O-Hexa-Hexa-Hex+Me+acetyl.

**Figure 10:**
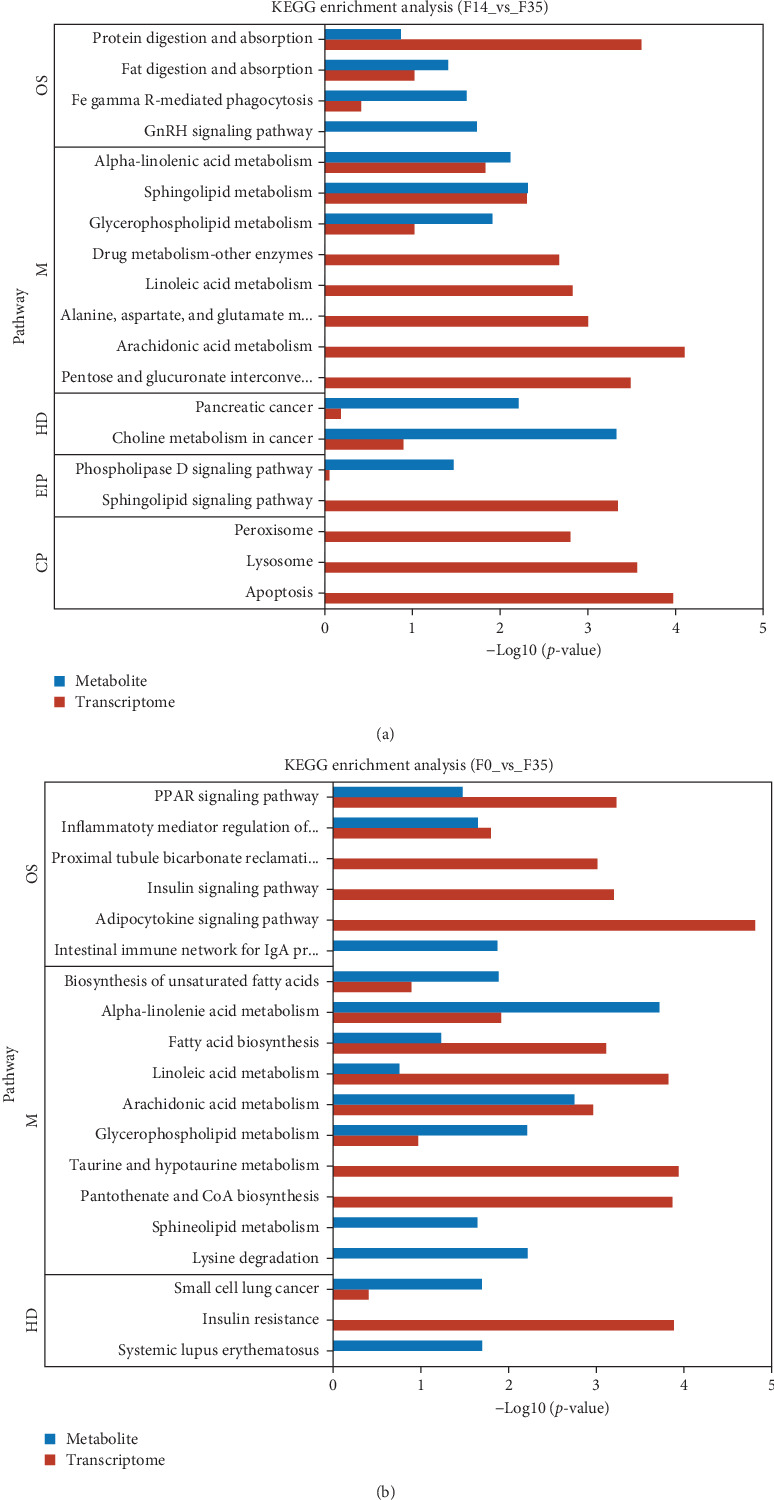
The integrative analysis of KEGG pathways based on transcriptomic and metabolomic predictions between *M. rosenbergii* fed F14 vs F35 groups (A) and F0 vs F35 groups (B). Pathways were considered significantly enriched when the *p*-value was below 0.05. F35: reference diet; F14 and F0: soy protein concentrate replaced 60% and 100% of the fish meal in diet F35, respectively.

Meanwhile, an integrative analysis of KEGG pathways was performed, with the analysis being based on transcriptomic and metabolomic predictions. In the F14 vs F35 groups, the predictions of common KEGG pathways based on transcriptomic and metabolomic were “pancreatic cancer”, “phospholipase D signaling pathway”, “protein digestion and absorption”, “fat digestion and absorption”, “alpha-linolenic acid metabolism”, “sphingolipid metabolism”, “choline metabolism in cancer”, “glycerophospholipid metabolism”, and “Fc gamma R-mediated phagocytosis” (Figure 10A). In the F0 vs F35 groups, the predictions of common KEGG pathways based on transcriptomic and metabolomic were “biosynthesis of unsaturated fatty acids”, “alpha-linolenic acid metabolism”, “small cell lung cancer”, “fatty acid biosynthesis”, “PPAR signaling pathway”, “linoleic acid metabolism”, “arachidonic acid metabolism”, “inflammatory mediator regulation of TRP channels”, and “glycerophospholipid metabolism” (Figure 10B). Of these, two KEGG pathways were shared, namely “alpha-linolenic acid metabolism” and “glycerophospholipid metabolism”.

## 4. Discussion

In the field of animal nutrition, the growth performance is understood to be a fundamental phenotypic indicator, used to evaluate the impact of dietary components on animal health and development [27]. A number of studies have demonstrated that FMC15 (replacing proportions of FM with cottonseed protein concentrate (CPC), with the final percentage of FM set at 150 g/kg) does not have a significant impact on weight gain, SGR, feed conversion ratio (FCR), or survival in *M. rosenbergii* [1]. However, the FMB15 (replacing proportions of FM with SBM, with the final percentage of FM set at 150 g/kg) has been observed to result in significant declines in weight gain, along with an increased FCR in *M. rosenbergii* [3, 19, 28]. These contrasting results suggest that the type of plant protein source plays a crucial role in organismal tolerance, even at the same substitution level. Furthermore, Soares et al. [29] reported that FMS8.8 (replacing proportions of FM with SPC, with the final percentage of FM set at 88 g/kg) diets did not have a negative effect on the growth performance of pacific white shrimp. However, the present study revealed that experimental diets comprising FM content less than 70 g/kg (F7 and F0 groups) impaired the growth performance of *M. rosenbergii*. Moreover, the current study revealed that experimental diets comprising FM content exceeding 140 g/kg (F35, F28, F21, and F14 groups) did not adversely affect the growth performance of *M. rosenbergii*, encompassing FBW, weight gain, SGR, and survival. In accordance with our findings, Chen et al. [30] observed that FMS15 (replacing proportions of FM with SPC, with the final percentage of FM set at 150 g/kg) had no adverse effects on FBW, weight gain, FCR, or survival in pacific white shrimp. The observed discrepancies between studies could be attributed to species-specific differences in tolerance to SPC, as well as variations in environmental conditions and dietary composition [10].

Furthermore, in the present study, significantly inhibited growth performance was observed in prawns fed diets with high levels of SPC replacement (FM inclusion levels below 140 g/kg, FM7 and FM0 groups). A similar trend of impaired growth performance has been reported for other plant protein sources, such as SBM [3, 28] and CPC [1], as well as in other aquatic species, such as pacific white shrimp [30], olive flounder [5], and large yellow croaker [2]. The decline in growth performance at elevated levels of SPC inclusion may be attributed to imbalances in the amino acid profile of SPC, particularly the reduced levels of lysine and methionine. Although equal amounts of methionine and lysine were added to all diets to meet the minimum growth requirements of *M. rosenbergii*, their suboptimal levels could still negatively affect growth. Further research is needed to determine the optimal levels of lysine and methionine for maximum growth, which will help refine and optimize feed formulations. The results of the present study indicate that *M. rosenbergii* exhibits a certain degree of tolerance to SPC inclusion levels of less than 211 g/kg, corresponding to FM replacement levels of up to 140 g/kg, without exhibiting any adverse effects on growth performance.

The effects of SPC on lipid deposition in aquatic animals appear to be species-specific, with observed outcomes ranging from increases and decreases to no significant effects. For instance, studies on *M. rosenbergii* (the present study), pacific white shrimp [30], pearl gentian groupers [16], olive flounder [5], and rice field eel (*Monopterus albus*) [31] reported no significant impact of SPC inclusion on whole-body crude lipid content. However, high levels of SPC replacement have been found to reduce whole-body lipid content in golden crucian carp [10]. This decline in lipid content was attributed to ANFs in SPC, which may impair intestinal health or directly inhibit nutrient utilization [32]. Conversely, the replacement of FM with SPC has been shown to increase whole-body crude lipid levels in pacific white shrimp [4, 30, 32]. In a similar vein, the impact of a SPC diet on crude protein of aquatic animal has shown varying results in the literature: inhibition were observed in golden crucian carp [10] and pacific white shrimp [30], no effects in *M. rosenbergii* (the present study) and pacific white shrimp [4, 32]. Intriguingly, even within the same species of pacific white shrimp, the results have been inconsistent. Whilst speculative, these discrepancies may arise from variations in experimental conditions, such as base feed composition, rearing environments, and genetic factors, which could influence body composition outcomes. In conclusion, the reduction in FM levels, accompanied by higher inclusion levels of SPC, had no discernible impact on the body composition of *M. rosenbergii* in the present study.

As the absorption and metabolism of nutrients is primarily facilitated through enterohepatic circulation [33], variations in growth performance are closely associated with the physiological condition of the intestine and hepatopancreas. The hepatopancreas serves as the primary energy storage organ and plays a critical role in hematopoiesis and energy metabolism in crustaceans. Previous studies have reported that replacing dietary FM with vegetable protein can lead to hepatopancreatic damage, impaired development, and a reduced hepatosomatic ratio [34]. The hepatopancreas is composed of hepatopancreatic tubules, which comprise various cell types, including ECs, BCs, absorptive cells (FCs), and RCs [4, 35]. Of these, BCs are responsible for detoxification and are known to exhibit swelling under conditions of oxidative stress. In pacific white shrimp, the ingestion of high plant-protein diets has been demonstrated to induce the shedding of the hepatopancreatic basement membrane, the enlargement of the tubular lumen, and the swelling of BCs, leading to the deformation and disorganization of the hepatopancreatic tubules [36, 37]. In a similar manner, *M. rosenbergii* fed diets with low FM levels exhibited a significant decrease in BCs abundance and an increase in vacuolation [19]. Consistent with these findings, the present study observed BCs swelling and a reduction in BCs abundance in the F14, F7, and F0 groups, indicating the occurrence of oxidative stress. Moreover, earlier studies have indicated that the more prevalent ECs could differentiate into RCs, which helps shrimp to absorb lipid and store it in the form of glycogen that can be released during molting and reproduction [4]. In the present study, the observed decrease in RCs abundance with increasing SPC levels is indicative of impaired nutrient utilization. The findings of this study provide clear evidence that elevated levels of dietary SPC, corresponding to reduced FM inclusion, have detrimental effects on the health of the hepatopancreas in *M. rosenbergii*.

The intestine plays a critical role in nutrient absorption, immune defense, and maintaining the overall health of crustaceans [38]. Previous studies have shown that the substitution of FM with plant proteins can impair intestinal barrier function, a phenomenon often attributed to the presence of ANFs. Such impairments can reduce nutrient utilization, which can result in compromised growth performance and feed efficiency in fish and crustaceans [10, 39]. Specifically, soybean agglutinin, for example, has been shown to bind to the intestinal mucosa, resulting in the destruction of brush border structures [40]. Soya-saponins have also been found to disrupt the tight junctions of intestinal cells, accelerate the proliferation and apoptosis of distal intestinal epithelial cells, and induce enteritis [15]. In addition, *β*-conglycinin has been reported to cause partial shedding of intestinal villi and separation of epithelial cells from the lamina propria [41]. In the case of *M. rosenbergii*, histological and ultrastructural analyses using hematoxylin and eosin (HE) staining and transmission electron microscopy have revealed that replacing FM with SBM significantly impairs intestinal morphology, including damage to villi and the basal membrane [3, 19]. However, the present study demonstrated that varying levels of SPC inclusion had no significant effects on intestinal fold height, mucosal thickness, or muscular thickness in *M. rosenbergii*. Our findings are consistent with those of previous studies reporting that the replacement of dietary FM with SPC did not affect distal intestinal histology in longfin yellowtail (*Seriola rivoliana*) [42], yellowtail kingfish (*Seriola lalandi*) [43], and golden crucian carp [10]. The findings of this study demonstrate that a reduction in FM levels, accompanied by an increase in SPC levels, exerts no discernible effect on the intestinal morphology of *M. rosenbergii*.

Oxidative stress induced by plant protein diets has been identified as a critical factor contributing to diminished growth performance in aquatic animals [19]. The SOD and GSH-PX enzymes are involved in the process of neutralizing excess free radicals, thereby mitigating oxidative damage in crustaceans [44]. Conversely, MDA functions as a biomarker of lipid peroxidation, reflecting the extent of cellular oxidative damage [45]. Furthermore, T-AOC is a comprehensive index of the body's antioxidant defense, encompassing both enzymatic and nonenzymatic antioxidants [25]. As demonstrated in previous research, the feeding of *M. rosenbergii* with FMC15 diet did not have a significant effect on the MDA content or the activities of CAT and SOD in the hemolymph [1]. In a similar manner, the present study established that a diet comprising 140 g/kg FM substituted by SPC exerted no substantial influence on MDA content, nor on the activities of SOD, GSH-PX, or T-AOC in *M. rosenbergii*. Furthermore, the present study demonstrated that the F0 treatment resulted in an increase in MDA content and a significant decrease in GSH-PX activity in comparison with the F35 treatment. These findings suggest that when the level of SPC in prawn feed exceeds the animal's tolerance threshold, it leads to impaired AOC. It is well established that ANFs present in plant proteins can reduce the AOC of aquatic animals. For instance, glycinin has been reported to suppress the expression of antioxidant enzyme-related genes, thereby decreasing the AOC of Jian carp (*C. carpio* var. Jian) [46]. Soy saponins have been shown to induce a significant, dose-dependent reduction in AOC [15], and *β*-conglycinin has been observed to cause oxidative damage in golden crucian carp [13]. Given the established association between ANFs and oxidative stress in fish, it can be hypothesized that these factors also contribute to oxidative stress in prawns. The results of this study demonstrate that *M. rosenbergii* can tolerate diets containing a minimum of 140 g/kg FM, equivalent to a maximum of 211 g/kg SPC, without any adverse effects on antioxidant properties.

Crustaceans primarily rely on innate immunity to defend against pathogens and other harmful agents [47]. Since key indicators of the innate immune system include ACP, AKP, and NO [48], the present study, therefore, measured changes in these immune-related parameters to evaluate the effect of varying SPC replacement levels on the immune performance of *M. rosenbergii*. Prior studies have indicated that elevated levels of plant protein, such as CPC, can compromise the immune function of *M. rosenbergii* [1]. In a similar manner, the present study observed that diets with high SPC inclusion (F0 group) significantly increased AKP activity and NO content in the hemolymph of *M. rosenbergii* compared to the F35 group. This finding is partially consistent with previous reports indicating that the activities of ACP and AKP in pacific white shrimp gradually increase with higher levels of SPC substitution [4, 32]. ANFs in plant proteins have been demonstrated to compromise immune function in fish. For instance, *β*-conglycinin and glycinin have been shown to induce inflammation in the intestine and liver, ultimately resulting in compromised immune capacity [41]. This knowledge provides a foundation for hypothesizing that analogous outcomes may be attained in crustaceans, where the elevated levels of SPC replacement may introduce ANFs that exceed the tolerance threshold of prawns, consequently suppressing their immune function. Overall, the findings of this study suggest that *M. rosenbergii* can tolerate diets with a minimum of 140 g/kg FM, equivalent to no more than 211 g/kg SPC inclusion, without exhibiting any adverse effects on immune-related parameters.

The present study performed transcriptomic and metabolomic analyses on the F35, F14, and F0 groups to ensure maximum variation in these indicators, based on differences in their growth performance. In order to gain a more profound comprehension of the metabolic responses associated with dietary SPC substitution in *M. rosenbergii*, a transcriptomics approach was employed. The OPLS-DA of annotated gene expression levels revealed that the gene expression profile and its functional structure in the hepatopancreas of prawns exhibited gradual changes with increasing levels of SPC replacing FM. These results are consistent with those of previous studies conducted on pearl gentian groupers [17, 18]. Furthermore, the VN plot demonstrated that approximately 85.79% of the total genes were shared among all three experimental groups (F35, F14, and F0). Our finding suggests that the observed variations among the groups are likely attributable not to the category of expressed genes but rather to the abundance of their expression. Among the DEGs, LOC136856310 (encoding sulfinoalanine decarboxylase) was identified as a shared DEG across all treatment groups. Sulfinoalanine decarboxylase is a critical enzyme involved in the biosynthesis of taurine through the biochemical conversion of cystine [49]. Previous research demonstrated that the expression of sulfinoalanine decarboxylase was significantly upregulated in the liver and intestine of Atlantic salmon (*Salmo salar*) fed 30% SBM compared to 0% SBM [50]. In a manner that is partially consistent with these findings, the present study observed that the expression of sulfinoalanine decarboxylase was initially significantly downregulated and then markedly upregulated as SPC substitution levels increased. This phenomenon can be ascribed to the diminished cystine content of the F14 diet, which consequently diminished taurine biosynthesis via cystine. Given the established fact that animal-derived proteins are rich in taurine while plant-derived proteins lack this compound [51], it can be hypothesized that although the cystine content in the F0 diet was comparable to that in the F35 diet, the increased demand for taurine in prawns fed F0 diet led to enhanced biosynthesis via this pathway. However, the taurine content of the experimental diets was not measured in this study, and thus, this hypothesis requires validation through further experiments.

Furthermore, the present study identified two KEGG pathways that were found to be significantly enriched based on the analysis of the transcriptome: “linoleic acid metabolism” and “arachidonic acid metabolism”. The Japanese flounder (*P. olivaceus*) juveniles fed a diet with FM partially replaced with SPC could significantly affect the content of omega-6 fatty acids in the liver, including linoleic acid [52]. In addition, the present experiments demonstrated that the level of SPC added to the feed can affect “linoleic acid metabolism” in prawns. Furthermore, the observed changes in the “arachidonic acid metabolism” pathway corroborate the findings of previous experiments, which identified the “arachidonic acid metabolism” pathway as the most significant KEGG pathway when comparing animal-based and soybean-based protein diets in rats [53]. Linoleic acid can be converted to other biologically active omega-6 polyunsaturated fatty acids, such as gamma-linolenic acid, dihomo-gamma-linolenic acid, and arachidonic acid, by desaturation and elongation, which may be the reason why “linoleic acid metabolism” and “arachidonic acid metabolism” were affected simultaneously in this experiment. In summary, increasing levels of SPC substitution and the consequent reduction in FM levels led to notable changes in the gene expression profile of *M. rosenbergii*. These changes were primarily associated with key metabolic pathways, including “linoleic acid metabolism” and “arachidonic acid metabolism”.

In accordance with the transcriptomic results, the structure of metabolite profiles in the hepatopancreas, as revealed by metabolomics, exhibited gradual changes with increasing levels of SPC replacing FM. These differences were primarily attributed to variations in metabolite abundance rather than differences in metabolite species. In a previous study on *M. rosenbergii*, diets containing SBM predicted a higher abundance of metabolites involved in the “primary bile acid biosynthesis” pathway [19]. In a similar manner, the present study identified 27-Deoxy-5B-Cyprinol as one of the VIP metabolites, which is associated with the “primary bile acid biosynthesis” pathway [54]. This enrichment may provide a rationale for the significant impact of the “primary bile acid biosynthesis” pathway across all dietary treatments. Moreover, evidence from previous research has shown that the addition of digested soybean protein to culture media significantly impacted the “glutathione metabolism” pathway in *Bifidobacterium animalis* [55]. In addition, elevated dietary plant protein content has been shown to significantly alter the “glutathione metabolism” pathways in both grass carp (*Ctenopharyngodon idellus*) and Atlantic salmon (*S. salar*) [56, 57]. In accordance with these observations, the present study noted a substantial enrichment of the “glutathione metabolism” pathway in prawns fed SPC-based diets. In addition to these metabolic effects, plant protein diets have been reported to suppress immune function by inhibiting apoptosis in amur sturgeon (*Acipenser schrenckii*) and turbot (*Scophthalmus maximus*) [58, 59]. Furthermore, the replacement of FM with SBM significantly influenced metabolites in the “purine metabolism” pathway in mirror carp (*C. carpio* Songpu) [20]. In a similar manner, the present study observed that SPC, derived from SBM, significantly affected the “autophagy-other”, “autophagy-animal”, and “purine metabolism” pathways in *M. rosenbergii*. Overall, as the level of SPC substitution increased (the level of FM decreased), substantial alterations in the metabolite profile structure of *M. rosenbergii* were observed. These changes were primarily associated with key metabolic pathways, including “glutathione metabolism”, “kaposi sarcoma-associated herpesvirus infection”, “autophagy-other”, “autophagy-animal”, “primary bile acid biosynthesis”, and “purine metabolism”.

The present study demonstrated that the LOC136856310 (encoding sulfinoalanine decarboxylase) was negatively corrected with EPA, arachidonoyl dopamine, 8Z,11Z-eicosadienoic acid, and vitamin E nicotinate. These are all significant lipids in FM [60]. A considerable body of research has evidenced the function of taurine in the modulation of lipid metabolism in aquatic animals [61]. The present study posits the hypothesis that sulfinoalanine decarboxylase-regulated taurine metabolism may also affect the metabolism of lipids in prawn. Furthermore, both metabolomic and transcriptomic results showed significant alterations in the “alpha-linolenic acid metabolism” and “glycerophospholipid metabolism”. Alpha-linolenic acid is a precursor for EPA and docosahexaenoic acid (DHA), which have been shown to play a key role in maintaining fish health through their participation in cell synthesis, neural tissue function and development, antioxidant ability, and immune system function [62]. It can be hypothesized that the alteration of “alpha-linolenic acid metabolism” induced by replacing FM with SPC may be an important cause of the altered immune and antioxidant properties of prawns. However, this is merely a conjecture that requires further experimentation to verify. In agreement with the results of the present study, the low FM diet significantly affected the “glycerophospholipid metabolism” in pacific white shrimp and *Oncorhynchus mykiss* [63, 64]. Reports had pointed out that the “glycerophospholipid metabolism” pathway may play a regulatory role in the levels of lipid metabolism and body inflammation, and that it may also stimulate an animal's ability to resist external stimuli [65]. It can be deduced that the modification of “glycerophospholipid metabolism” may be a further significant factor contributing to the inflammatory response exhibited by prawns in the present experiment. In summary, a decrease in feed FM levels or an increase in SPC replacement levels resulted in substantial alterations to the metabolism of “alpha-linolenic acid metabolism” and “glycerophospholipid metabolism”. Consequently, these alterations indirectly influenced the physiological functions of the *M. rosenbergii*, including their inflammatory responses.

## 5. Conclusion

The present study demonstrated the following key findings: (1) based on growth performance, dietary FM level for *M. rosenbergii* could be reduced to 140 g/kg by using SPC as a sole substitute. (2) Elevated levels of dietary SPC have been shown to have a detrimental effect on the hepatopancreas health, AOC, and immune properties of *M. rosenbergii*. (3) A comprehensive analysis of both metabolomic and transcriptomic data sets revealed substantial perturbations in the pathways associated with “alpha-Linolenic acid metabolism” and “glycerophospholipid metabolism”. These findings emphasized that SPC can serve as a viable plant-based protein source for replacement of FM in prawn diets when used under appropriate dietary strategies. Furthermore, this study provides valuable insights into the metabolic adaptations of *M. rosenbergii* to diets based on SPC, which contributes to the development of sustainable and nutritionally balanced aquafeeds.

## Figures and Tables

**Table 1:** Proximate composition (g/kg) of the feed ingredients.

Ingredient	Dry matter	Crude protein^1^	Crude lipid^1^	Ash^1^
Fish meal	926	667	78	176
Poultry by-product meal	929	610	128	187
Soy protein concentrate	937	663	26	46
Soybean meal	890	441	13	65
Wheat flour	883	117	5	2

^1^Crude protein, crude lipid, and ash are expressed as the situation stored in air (*n* = 2).

**Table 2:** Formulation (g/kg) and proximate composition (g/kg) of the test diets.

Ingredient	F35^1^	F28^1^	F21^1^	F14^1^	F7^1^	F0^1^
Fish meal	350	280	210	140	70	0
Soy protein concentrate	0	70	141	211	282	352
Poultry by-product meal	100	100	100	100	100	100
Soybean meal	166	166	166	166	166	166
Wheat flour	220	220	220	220	220	220
Choline chloride	2	2	2	2	2	2
Phospholipid	10	10	10	10	10	10
Microcrystalline cellulose	37	33	28	24	19	15
Cholesterol	5	5	5	5	5	5
Antioxidant	1	1	1	1	1	1
Sodium carboxymethylcellulose	30	30	30	30	30	30
Ca (H_2_PO_4_)_2_	20	20	20	20	20	20
DL-Met	2	2	2	2	2	2
L-Lysine	5	5	5	5	5	5
Vitamin and mineral premix^2^	30	30	30	30	30	30
Soybean oil	11	13	15	17	19	21
Fish oil	11	13	15	17	19	21
Dry matter	886	901	899	886	889	899
Crude protein^3^	391	399	390	382	383	383
Crude lipid^3^	76	74	73	73	73	73
Ash^3^	122	115	107	98	88	81

^1^F35: reference diet; F28, F21, F14, F7, and F0: soy protein concentrate replaced 20%, 40%, 60%, 80%, and 100% of the fish meal in diet F35, respectively.

^2^Vitamin and mineral premix (per kg diet): vitamin A, 8000 IU; vitamin D_3_, 2000 IU; vitamin E, 100 mg; vitamin K_3_, 7.5 mg; vitamin B_1_, 15 mg; vitamin B_2_, 15 mg; vitamin B_6_, 12.5 mg; vitamin B_12_, 0.05 mg; D-biotin, 0.25 mg; D-calcium pantothenate, 40 mg; folic acid, 5 mg; niacinamide, 50 mg; vitamin C, 140 mg; inositol, 120 mg; ethoxyquin, 5 mg; FeSO_4_, 40 mg; CuSO_4_·5H_2_O, 25 mg; MnSO_4_·4H_2_O, 10 mg; ZnSO_4_, 100 mg; MgSO_4_·7H_2_O, 200 mg; CoCO_3_, 0.35 mg; KI, 0.05 mg; Na_2_SeO_3_, 0.3 mg.

^3^Contents of crude protein, crude lipid, and ash reflect the situation of the feed ingredients in natural storage.

**Table 3:** Amino acid content (g/kg) of the test diets.

Amino acid	F35^1^	F28^1^	F21^1^	F14^1^	F7^1^	F0^1^
Aspartic	33.40	34.90	36.90	35.30	37.90	39.70
Threonine	14.60	15.00	14.90	13.50	14.10	14.40
Serine	14.30	15.70	15.90	15.60	16.30	17.70
Glutamate	58.10	62.00	64.30	64.60	69.50	73.80
Glycine	22.80	22.40	22.50	19.30	19.20	18.60
Alanine	23.20	23.50	23.00	20.70	20.20	19.40
Cystine	4.40	3.80	4.10	3.80	4.30	4.50
Valine	17.30	16.80	17.50	15.90	17.10	17.00
Methionine	8.50	7.80	7.50	4.80	5.50	4.80
Isoleucine	15.50	15.70	16.30	15.50	16.40	16.70
Leucine	27.40	27.90	28.70	27.40	28.60	29.20
Tyrosine	10.30	10.30	10.80	10.10	10.90	11.20
Phenylalanine	15.70	16.30	16.60	16.40	17.70	18.30
Lysine	28.20	27.60	27.90	25.00	25.00	24.70
Histidine	9.90	10.80	10.20	9.00	8.90	9.10
Arginine	21.60	23.10	24.70	23.80	25.20	26.10
Proline	18.90	19.70	20.00	20.60	20.60	20.90
Total	344.20	353.20	361.90	341.30	357.40	366.10

*Note:* Amino acids are expressed as the situation of the ingredients stored in air (*n* = 2).

^1^F35: reference diet; F28, F21, F14, F7, and F0: soy protein concentrate replaced 20%, 40%, 60%, 80%, and 100% of the fish meal in diet F35, respectively.

**Table 4:** Survival, weight gain, and specific growth rate of *M. rosenbergii*.

Diet	IBW^1^ (g)	FBW^1^ (g)	Weight gain (g)	SGR^1^ (%/day)	Survival (%)
F35	0.22 ± 0.01	2.46 ± 0.15^a^	2.24 ± 0.15^a^	4.45 ± 0.10^a^	70.00 ± 8.00
F28	0.22 ± 0.01	2.36 ± 0.10^a,b^	2.14 ± 0.10^a,b^	4.38 ± 0.03^a,b^	77.33 ± 7.57
F21	0.22 ± 0.00	2.37 ± 0.33^a,b^	2.15 ± 0.33^ab^	4.38 ± 0.27^a,b^	75.33 ± 4.16
F14	0.22 ± 0.01	2.12 ± 0.12^a,b^	1.89 ± 0.11^a,b^	4.15 ± 0.10^a,b^	72.67 ± 3.06
F7	0.22 ± 0.00	1.98 ± 0.42^b^	1.76 ± 0.42^b^	4.00 ± 0.45^b^	80.00 ± 7.21
F0	0.22 ± 0.00	1.97 ± 0.10^b^	1.75 ± 0.09^b^	4.04 ± 0.09^a,b^	72.00 ± 12.17

*Note:* F35: reference diet; F28, F21, F14, F7, and F0: soy protein concentrate replaced 20%, 40%, 60%, 80%, and 100% of the fish meal in diet F35, respectively. Data are presented as mean ± SD (*n* = 4). The superscripts present the results of Duncan's test between F35, F28, F21, F14, F7, and F0 (small letters). The data in the same column with different superscripts are significantly different (*p* < 0.05).

^1^FBW, final body weight; IBM, initial body weight; SGR, specific growth rate.

**Table 5:** Hepatosomatic index and body composition of *M*. *rosenbergii*.

Diet	HSI^1^ (%)	Moisture (%)	Crude protein^2^ (%)	Crude lipid^2^ (%)	Ash^2^ (%)
F35	3.64 ± 0.73	76.97 ± 1.47	15.65 ± 0.93	1.05 ± 0.22^a,b^	4.62 ± 0.37^a,b^
F28	3.85 ± 0.68	77.08 ± 0.43	15.56 ± 0.35	0.87 ± 0.13^b^	4.82 ± 0.18^a,b^
F21	3.75 ± 0.85	77.23 ± 1.12	15.38 ± 0.86	1.02 ± 0.21^a,b^	4.75 ± 0.29^a,b^
F14	3.92 ± 0.70	77.38 ± 0.70	15.53 ± 0.61	1.11 ± 0.18^a,b^	4.45 ± 0.15^b^
F7	3.91 ± 0.82	77.27 ± 0.59	15.04 ± 0.37	1.03 ± 0.07^a,b^	5.00 ± 0.24^a^
F0	3.73 ± 0.87	76.47 ± 0.24	15.60 ± 0.28	1.17 ± 0.19^a^	4.77 ± 0.40^a,b^

^1^HSI, hepatosomatic index.

^2^Crude protein, crude lipid, and ash are expressed on a wet weight basis. F35: reference diet; F28, F21, F14, F7, and F0: soy protein concentrate replaced 20%, 40%, 60%, 80%, and 100% of the fish meal in diet F35, respectively. Data are presented as mean ± SD (*n* = 4). The superscripts present the results of Duncan's test between F35, F28, F21, F14, F7, and F0 (small letters). The data in the same column with different superscripts are significantly different (*p* < 0.05).

**Table 6:** Fold height, mucosal thickness, and muscular thickness of intestinal sections of *M. rosenbergii*.

Diet	Fold height (μm)	Mucosal thickness (μm)	Muscular thickness (μm)
F35	52.81 ± 25.47	39.56 ± 7.36^a,b^	12.52 ± 1.68
F28	32.52 ± 11.81	55.19 ± 18.10^a,b^	13.20 ± 1.85
F21	28.56 ± 7.38	62.09 ± 5.14^a^	14.22 ± 3.08
F14	34.27 ± 14.16	47.36 ± 8.73^a,b^	14.97 ± 2.41
F7	39.59 ± 13.71	53.91 ± 10.14^a,b^	11.10 ± 3.51
F0	32.15 ± 1.84	33.31 ± 13.85^b^	10.76 ± 2.84

*Note:* F35: reference diet; F28, F21, F14, F7, and F0: soy protein concentrate replaced 20%, 40%, 60%, 80%, and 100% of the fish meal in diet F35, respectively. Data are presented as mean ± SD (*n* = 4). The superscripts present the results of Duncan's test between F35, F28, F21, F14, F7, and F0 (small letters). The data in the same column with different superscripts are significantly different (*p* < 0.05).

**Table 7:** Antioxidant and phosphatase enzymes activity, contents of malondialdehyde and nitric oxide in hemolymph of *M. rosenbergii*.

Diet	T-AOC^1^ (mM)	SOD^1^ (U/mL)	GSH-PX^1^ (U/mL)	MDA^1^ (nmol/mL)	AKP (U/100 mL)	ACP^1^ (U/100 mL)	NO^1^ (nmol/mL)
F35	0.91 ± 0.22	41.00 ± 0.88	1206.96 ± 225.31^a^	17.89 ± 1.41^a,b^	0.38 ± 0.08^b^	2.19 ± 0.27	4.43 ± 1.00^b^
F28	0.90 ± 0.30	41.84 ± 1.73	1291.89 ± 298.68^a^	18.88 ± 3.10^a,b^	0.31 ± 0.26^b^	2.74 ± 0.88	4.22 ± 1.52^b^
F21	0.99 ± 0.25	42.58 ± 1.48	1017.60 ± 151.28^a,b^	17.43 ± 3.93^a,b^	0.24 ± 0.10^b^	2.16 ± 0.44	4.33 ± 1.70^b^
F14	0.85 ± 0.07	42.43 ± 2.28	994.78 ± 113.88^a,b,c^	16.59 ± 0.84^b^	0.29 ± 0.31^b^	2.29 ± 0.61	4.64 ± 1.84^b^
F7	0.95 ± 0.16	42.01 ± 1.45	693.94 ± 98.59^c^	19.52 ± 2.10^ab^	0.40 ± 0.45^b^	2.65 ± 1.35	4.11 ± 1.15^b^
F0	0.75 ± 0.11	40.96 ± 0.77	733.91 ± 93.25^b,c^	21.46 ± 1.95^a^	0.92 ± 0.29^a^	2.98 ± 0.66	8.22 ± 1.49^a^

*Note:* F35: reference diet; F28, F21, F14, F7, and F0: soy protein concentrate replaced 20%, 40%, 60%, 80%, and 100% of the fish meal in diet F35, respectively. Data are presented as mean ± SD (*n* = 4). The superscripts present the results of Duncan's test between F35, F28, F21, F14, F7, and F0 (small letters). The data in the same column with different superscripts are significantly different (*p* < 0.05).

^1^T-AOC, total antioxidant capacity; SOD, superoxide dismutase; GSH-PX, glutathione peroxidase; MDA, malondialdehyde; AKP, alkaline phosphatase; ACP, acid phosphatase; NO, nitric oxide.

## Data Availability

The data that support the findings of this study are available from the corresponding author upon reasonable request.
